# Glycolytic shift during West Nile virus infection provides new therapeutic opportunities

**DOI:** 10.1186/s12974-023-02899-3

**Published:** 2023-09-27

**Authors:** Patricia Mingo-Casas, Ana-Belén Blázquez, Marta Gómez de Cedrón, Ana San-Félix, Susana Molina, Estela Escribano-Romero, Eva Calvo-Pinilla, Nereida Jiménez de Oya, Ana Ramírez de Molina, Juan-Carlos Saiz, María-Jesús Pérez-Pérez, Miguel A. Martín-Acebes

**Affiliations:** 1 Present Address: Department of Biotechnology, Instituto Nacional de Investigación y Tecnología Agraria y Alimentaria, Consejo Superior de Investigaciones Científicas (INIA-CSIC), 28040 Madrid, Spain; 2 Molecular Oncology Group, IMDEA Food Institute, CEI UAM + CSIC, 28049 Madrid, Spain; 3 Instituto de Quimica Medica (IQM), CSIC, 28006 Madrid, Spain

**Keywords:** West Nile virus, Glycolysis, Immunometabolism, Neuroinflammation

## Abstract

**Background:**

Viral rewiring of host bioenergetics and immunometabolism may provide novel targets for therapeutic interventions against viral infections. Here, we have explored the effect on bioenergetics during the infection with the mosquito-borne flavivirus West Nile virus (WNV), a medically relevant neurotropic pathogen causing outbreaks of meningitis and encephalitis worldwide.

**Results:**

A systematic literature search and meta-analysis pointed to a misbalance of glucose homeostasis in the central nervous system of WNV patients. Real-time bioenergetic analyses confirmed upregulation of aerobic glycolysis and a reduction of mitochondrial oxidative phosphorylation during viral replication in cultured cells. Transcriptomics analyses in neural tissues from experimentally infected mice unveiled a glycolytic shift including the upregulation of hexokinases 2 and 3 (*Hk2* and *Hk3*) and pyruvate dehydrogenase kinase 4 (*Pdk4*). Treatment of infected mice with the Hk inhibitor, 2-deoxy-D-glucose, or the Pdk4 inhibitor, dichloroacetate, alleviated WNV-induced neuroinflammation.

**Conclusions:**

These results highlight the importance of host energetic metabolism and specifically glycolysis in WNV infection in vivo. This study provides proof of concept for the druggability of the glycolytic pathway for the future development of therapies to combat WNV pathology.

**Supplementary Information:**

The online version contains supplementary material available at 10.1186/s12974-023-02899-3.

## Introduction

The COVID-19 pandemic has evidenced the high impact of viral infections on health, the economy, and society, and the urgent need for improved antiviral therapies. The analysis of the pathophysiology of SARS-CoV-2 infection also highlighted that the pathologies associated with viral diseases are not only the direct result of to the viral multiplication. Indeed, the host immune response and inflammatory-associated pathologies play important roles in the clinical conditions of infected patients [1]. Accordingly, it is mandatory to continue exploring novel targets for therapeutic interventions against emerging viral diseases. Arthropod-borne viruses (arboviruses) constitute an important group of emerging viral pathogens. Factors such as climate warming, globalization of travel and trade, urbanization, or changes in land use, favor vector expansion contributing to arbovirus dissemination and incidence [2, 3]. One of the most distributed medically relevant arboviruses is West Nile virus (WNV), a mosquito-borne flavivirus responsible for recurrent outbreaks of meningitis and encephalitis, affecting humans and horses [4]. Although preventive equine vaccines against WNV are available, no vaccines for humans or specific drugs are licensed to prevent or treat the disease.

Considering that virus infection rewires host metabolism and that immune system components undergo metabolic shifts as part of the host response against viral infections, targeting bioenergetics and immunometabolism may provide novel opportunities for therapeutic treatments [5, 6]. Glycolysis, one of the major bioenergetic pathways, is the catabolic pathway that converts glucose into pyruvate and can fuel viral replication and immunity, so it has been long associated with viral infections since the changes in glycolysis were first associated with poliovirus infection [7]. Currently, the relevance of glycolysis in viral infections has been reported for different viruses, including some Flaviviruses [8–11]. In addition, glycolysis is also important for the induction of the immune response and inflammation, thus making this pathway an interesting target for therapeutics [10, 12]. In the specific case of WNV, evidence from proteomics studies supported the differential expression of energy metabolism-related cellular factors during the infection in cultured cells, reporting the overexpression of an isoform of pyruvate kinase 3, a key enzyme of the glycolytic pathway [13]. However, to our knowledge, host bioenergetics during WNV infection in vivo and, more importantly, its potential for therapeutic development has not been explored so far for this pathogen.

In this work, we have analyzed the impact on host bioenergetics during WNV infection identifying glycolysis as a key metabolic pathway. Our results indicate the upregulation of glycolysis upon WNV infection in both cell culture and target tissues in a mouse model of WNV infection, providing a proof of concept for the druggability of this metabolic pathway to alleviate WNV-induced neuroinflammation. These results settle the basis for the development of future therapies to combat WNV pathology based on metabolic modulators.

## Materials and methods

### Cells, viruses, and infections

Virus infections were performed on Vero ATCC CCL-81 or Neuro-2a CCL-131 cells using WNV New York 99 strain [14] (GenBank: KC407666.1). Virus was titrated in Vero cells by standard plaque assay in semisolid agar medium. Procedures for cell culture, infections and virus titration have been previously described [15–17]. Cells were infected at a multiplicity of infection (MOI) of 1 plaque forming unit (PFU)/cell, incubated 1 h at 37 °C, and then viral inoculum was removed and, when required, replaced by fresh medium containing the drugs. AL-429 was synthetized by us as described [18]. Sodium oxamate, sodium dichloroacetate (DCA), and 2-deoxy-D-glucose (2-DG) were purchased from Sigma (San Luis, MO, USA). Stock solutions were prepared in dimethyl sulfoxide (DMSO), which was also used as control vehicle. For glucose depletion studies, cells were grown in Dulbecco’s Modified Eagle medium without D-Glucose (Ref. 11966-025 Gibco, Fisher, Waltham, MA, USA). Cellular ATP content was determined in uninfected cells treated in parallel using CellTiter-Glo Luminescent Cell Viability Assay (Promega). WNV single-round reporter virus particles (RVPs) were produced by the complementation in *trans* of a subgenomic reporter replicon [19] using a pcDNA 3.1 + vector expressing WNV structural proteins C, prM, and E of WNV Novi Sad/12 (GenBank accession number KC407673.1), as described [20]. Plasmids were then transfected into human embryonic kidney HEK 293T cells (ATCC CRL-11268) with DharmaFECT kb DNA transfection reagent (Dharmacon, Lafayette, CO, USA) according to manufacturer’s instruction. The RVPs were collected from the supernatants at 48 h post-transfection to infect Vero cells monolayers for real-time bioenergetic analyses.

### Animal experiments

Experimental infections in mice were performed in the biosafety level 3 (BSL-3) facilities at Centro de Investigación en Sanidad Animal, Instituto Nacional de Investigación y Tecnología Agraria y Alimentaria (CISA, INIA-CSIC). Six-week-old Hsd:ICR(CD-1) female mice (Inotiv) were used. Mice were infected intraperitoneally (i.p.) with 10^4^ PFU of WNV New York 99 strain [14] (GenBank: KC407666.1) in 200 µL of Minimum Essential Medium Eagle (Corning); mock-infected animals were also inoculated i.p. with 200 µL of culture media. In the case of drug treatments, drugs or vehicle (saline buffer, 0.9% NaCl) were intraperitoneally administered at a dose of 200 mg/kg for DCA or 500 mg/kg in the case of 2-DG. Mice were daily injected (QD) from the first day of infection up to day 6 post-infection. Animals were kept with ad libitum access to food and water and were anesthetized under isoflurane and humanely killed at 7 days post-infection.

### Literature search and meta-analysis

A systematic literature review was performed following PRISMA 2020 guidelines (http://prisma-statement.org/?AspxAutoDetectCookieSupport=1). Two independent reviewers assessed the risk of bias and performed data extraction. Database searches in PubMed (https://pubmed.ncbi.nlm.nih.gov/) aimed to include all original, peer-reviewed studies in English. Pre-defined terms regarding WNV acute human infections with clinical data to obtain blood glucose, CSF glucose, and/or CSF/blood glucose ratio values were used. A flow diagram was generated to represent the number of records identified, screened, and included.

### Metabolic flux measurement

Real-time measurement of oxygen consumption rate (OCR) and extracellular acidification rate (ECAR) were monitored using Vero cells infected with RVPs, with the XFe extracellular flux analyzer (Agilent Seahorse Technologies, Santa Clara, CA, USA). Briefly, 10,000 Vero cells/well were plated into XF96 microplates for ECAR measurement, and 15,000 cells/well for OCR measurement. Prior to the analysis, media was changed to XF DMEM (Agilent Seahorse Technologies, Santa Clara, CA, USA) supplemented with 10 mM glucose (Agilent Seahorse Bioscience), 1 mM sodium pyruvate and 2 mM L-glutamine for OCR (readout of mitochondrial respiration) and to XF DMEM (Agilent Seahorse Technologies, Santa Clara, CA, USA) supplemented with 2 mM of glutamine for ECAR monitorization as an indirect readout of acidification by the production of lactate (aerobic glycolysis). For ECAR measurement the following sequential injections were performed: glucose (10 mM), oligomycin (2 µM), and 2-DG (75 mM). For OCR, sequential injection of oligomycin (2 µM), trifluoromethoxy carbonylcyanide phenylhydrazone (FCCP; 1.2 µM), and rotenone/antimycin A (0.5 µM) as previously described [21, 22].

### RNA-seq

Total RNA was extracted from tissue samples using Ribopure (Invitrogen). The cDNA library was constructed with TruSeq Stranded mRNA LT Sample Prep Kit (Illumina, San Diego, CA, USA) and sequencing was performed on a NovaSeq6000 100PE (Illumina, San Diego, USA) by Macrogen (Seoul, Korea). Raw reads quality was analyzed with FastQC to obtain phred quality score and trimmed with Trimmomatic [23] to remove adapter sequences. Reads were filtered with a 4-base pair (bp) sliding window and trimmed if the mean of the phred score was below 15. Finally, reads under 36 bp length were removed. Processed reads were mapped to mm10 reference genome with BowTie [24] and HISAT2 [25] and transcripts were assembled with StringTie [26]. The expression profile was then calculated for each gene as read count, PKM (Fragment per Kilobase of transcript per Million mapped reads), and TPM (Transcripts Per Kilobase Million). Differentially expressed genes (DEGs) analysis was performed using DESeq2 [27] and selected when |FC|> = 2 and nbinom Wald test raw *p*-value < 0.05. Each *p*-value was corrected by FDR (Benjamini–Hochberg) and variations in gene expression level were expressed as log_2_ of fold change (FC). Corrected DEGs (adjusted FDR *p*-value < 0.05) were also analyzed with web-based gProfiler (https://biit.cs.ut.ee/gprofiler/gost) for gene set enrichment per biological process (BP). Gene Set Enrichment Analysis (GSEA) [28, 29] of each pair of transcriptomic datasets was performed with GSEA software 4.2.3 by using Hallmark gene set database from Mouse MSigDB Collections [30]. The number of permutations was set to 1000 and gene symbols were not collapsed. Phenotype labels were set as WNV 3 d.p.i *vs* uninfected 3 d.p.i, WNV 7 dpi *vs* uninfected 7 dpi, and WNV 10 dpi *vs* uninfected 10 dpi. To identify specific metabolic genes, Reactome pathways [31] of glycolysis (R-MMU-70171), pyruvate metabolism and TCA cycle (R-MMU-71406), pentose phosphate pathway (R-MMU-71336) and respiratory electron transport (R-MMU-611105) were downloaded and used to obtain cross-matches with our corrected DEGs lists. Antiviral mechanism by IFN-stimulated genes (R-MMU-1169410) and signaling by interleukins (R-MMU-449147) pathway were interrogated to identify DEGs associated with antiviral immune response. RNA-seq cell type annotation was performed using CellKb (https://www.cellkb.com) by analyzing top 100 corrected by FDR DEGs in descending order of fold change and filtering by anatomical structure. Total scores for each unique, non-redundant cell type were selected for representation.

### Quantitative PCR

Tissue samples were homogenized in TRI-Reagent (Invitrogen) using a TissueLyserII equipment (Qiagen) and total RNA was extracted with Ribopure kit (Invitrogen). Virus load was determined by one-step reverse transcription (RT) followed by quantitative PCR (qPCR) as previously described [32] using a QuantStudio5 Real-time PCR system (Applied Biosystems). For gene expression analyses cDNA was synthesized using Biotools High Retrotranscriptase Starter Kit with Oligo dT (Biotools). The expression levels of *Ccl2*, *Cxcl10*, *Cxcl11*, and *TNF-α* were analyzed with Applied Biosystems™ TaqMan™ Array Mouse Immune Response (Applied Biosystems, Waltham, MA, USA), according to manufacturer indications, using *GAPDH* as endogenous control. The analysis of *Hk3* and *Slc16a3* expression relative to *GAPDH* was performed using PrimeTime Std qPCR Assays from Integrated DNA Technologies (Mm.PT.39a.1 for *GAPDH*, Mm.PT.58.9533034 for *Hk3* and Mm.PT.58.8171114 Mm.PT.58.43575827 for *Slc16a3*) as previously described [33].

### Data analysis

Data are presented as mean ± standard error of the mean (SEM). The number of biological replicates for tissue culture experiments or individual animals analyzed in each case is denoted by *n* in the figure legends. PRISM GraphPad 7.0 was used to determine the statistical significance of the data. Unpaired *t*-test was applied for comparisons between two groups using Welch’s correction in the case of different standard deviations. One-way ANOVA followed by Dunnett’s multiple comparison correction was applied in the case of comparison with a single control group. Two-way ANOVA and Sidak’s multiple comparison tests were performed for pairwise comparisons. In the case of non-parametric data, Kruskal–Wallis test followed by Dunn’s correction for multiple comparisons. DEGs *p*-value was corrected by FDR. *P < 0.05; **P < 0.01; ***P < 0.001; ****P < 0.0001.

## Results and discussion

### Literature search and meta-analysis for glucose levels in human patients infected with WNV

**Fig. 1:**
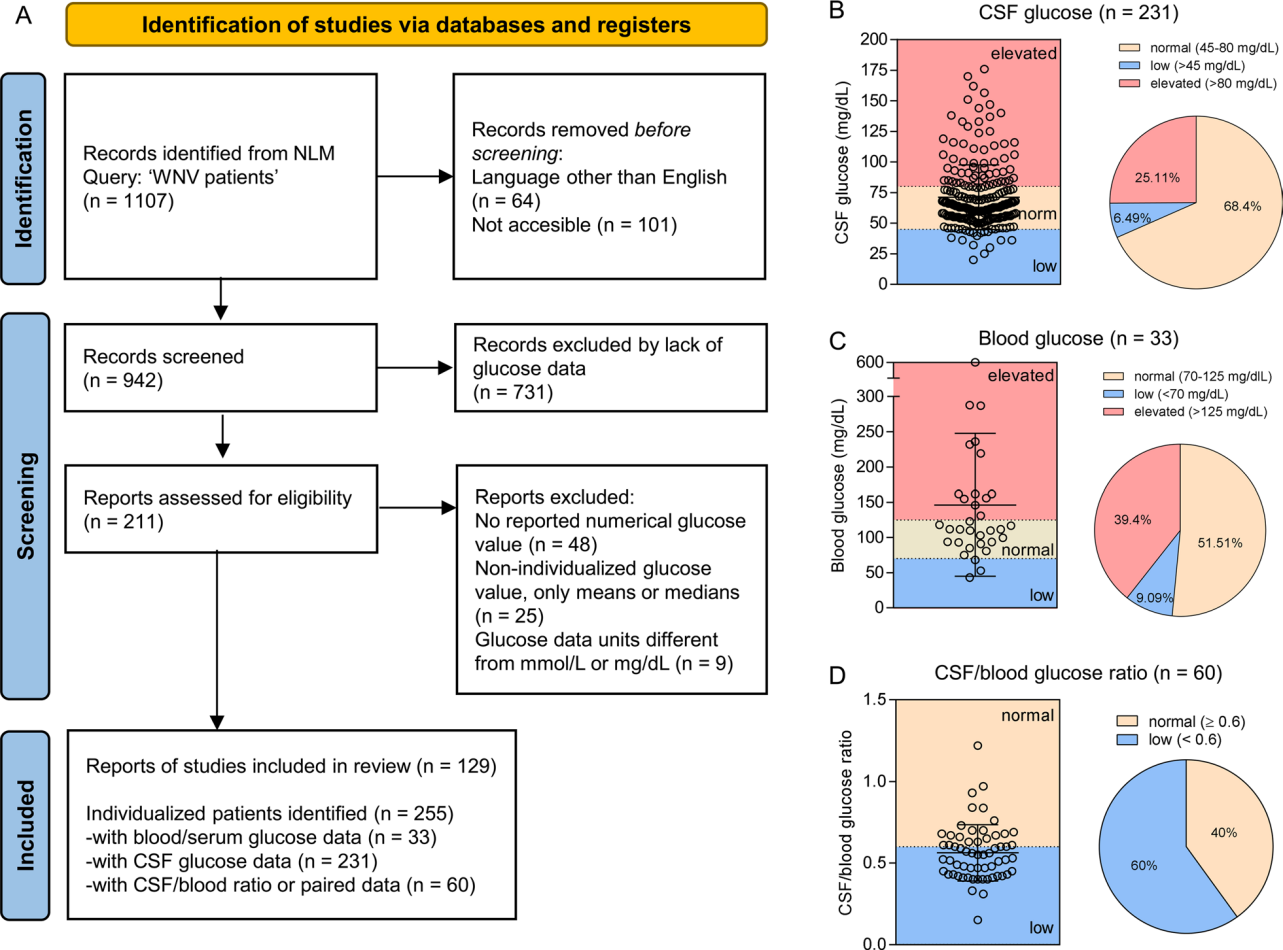
Literature search and meta-analysis for glucose levels in human patients infected with WNV. **A** Flowchart for the systematic literature search and meta-analysis of glucose levels in patients infected with WNV according to PRISMA guidelines. **B**–**D** Results from individualized patients identified in the literature search for **B** CSF glucose (*n* = 231); **C** blood glucose (*n* = 33); **D** CSF/blood glucose ratio (*n* = 60). Dashed lines denote normal values in healthy individuals (see text for details on reference values)

Glucose is one of the major energy sources and pathogen infections can alter glucose homeostasis in the central nervous system (CNS) [34]. To evaluate the impact of WNV infection on CNS glucose levels, a systematic literature search together with the respective meta-analysis was performed (Fig. 1A). Glucose levels from 129 studies covering a total of 255 patients were collected. Normal glucose levels in CSF lie between 45 and 80 mg/dL (2.5–4.4 mmol/L) and 70–125 mg/dL (3.9–6.9 mmol/L) in blood (https://www.who.int/data/gho/indicator-metadata-registry/imr-details/2380). Most WNV patients displayed normal glucose levels in the CSF (68.4%) with a mean glucose value of 71.18 ± 26.51 mg/dL (Fig. 1B) which is consistent with previous studies on patient cohorts [35, 36]. Similarly, the majority of the patients (51.51%) also displayed normal glycemia. A patient that developed diabetic ketoacidosis with a blood glucose level of 600 mg/dL as a consequence of the infection was identified in the study [37]. Excluding this outlier from the analyses, only a slightly elevated mean value of blood glucose of 132.10 mg/dL ± 61.63 was found (Fig. 1C). The glucose level in CSF under normal physiological conditions is proportional to the blood glucose level and corresponds to 60–70% of the concentration in blood [38], being the normal ratio of 0.6 [34]. When the CSF/blood glucose ratio was calculated for WNV patients, an important fraction (60%) exhibited a CSF/glucose ratio lower than 0.6, thus suggesting an effect of WNV infection on glucose homeostasis in the CNS (Fig. 1D).

### Bioenergetics of WNV infection in cultured cells

**Fig. 2:**
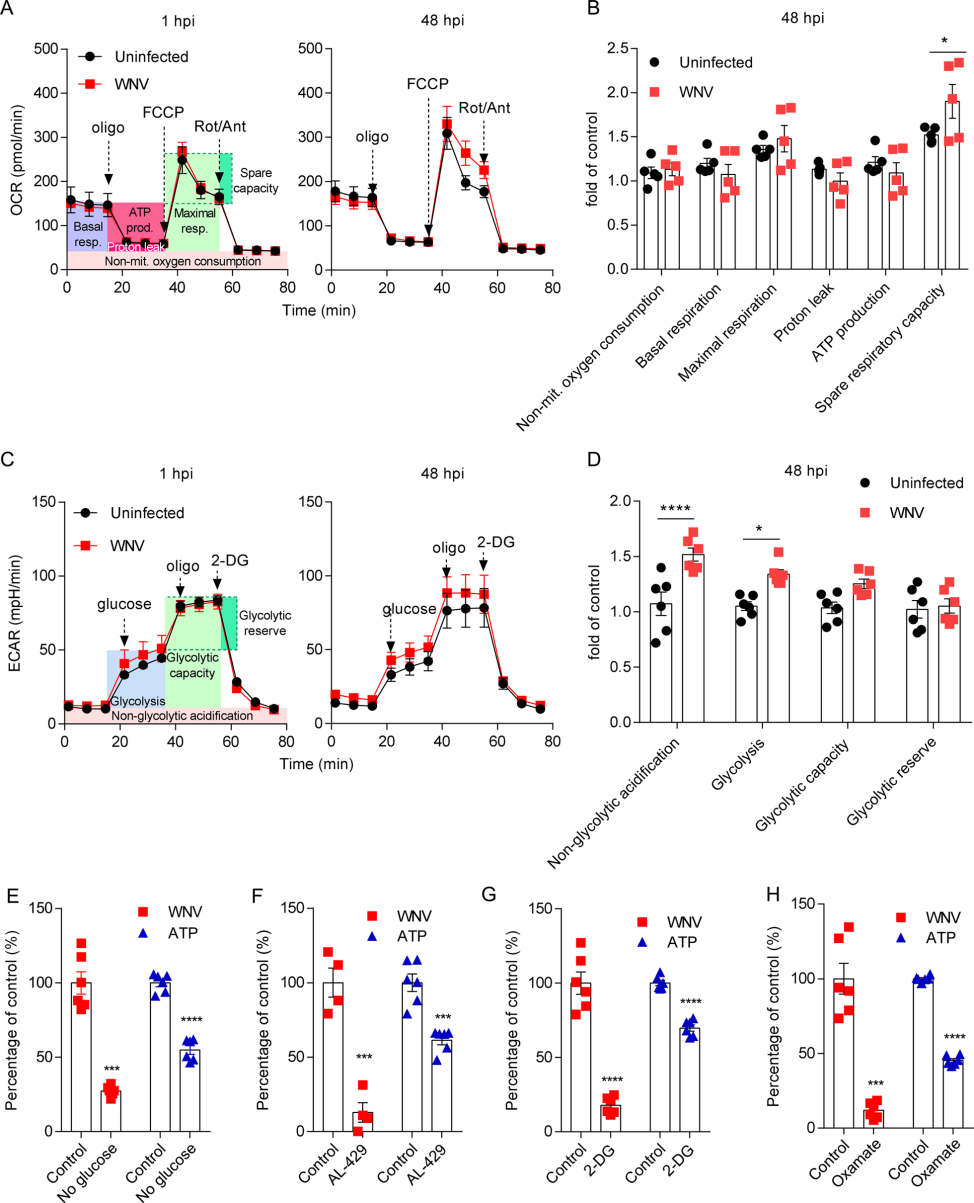
Bioenergetics of WNV infection in cultured cells. **A** Changes in mitochondrial respiration profile induced by WNV replication. Real-time monitorization of oxygen consumption rate (OCR) in Vero cells infected or not with WNV RVPs at 1 or 48 hpi (*n* = 5). Arrows indicate the time of injection of oligomycin, FCCP and rotenone/antimycin A. Respiratory parameters determined are indicated by colored areas. **B** Comparison of respiratory parameters determined by OCR measurement between control (uninfected) and infected cells with RVPs at 48 hpi. Fold change over uninfected at 1 hpi is represented. ***, *P* < 0.001 for two-way ANOVA and Sidak’s multiple comparisons test (*n* = 9). **C** Changes in glycolytic rate induced by WNV replication. Real-time monitorization of extracellular acidification rate (ECAR) was measured in Vero cells infected or not with WNV RVPs at 1 or 48 h pi (*n* = 6). Arrows indicate the time of injection of glucose, oligomycin and 2-DG. Glycolytic parameters determined are indicated by colored areas. **D** Comparison of glycolytic parameters determined by ECAR measurement between control (uninfected) and cell infected with SRIPs at 48 hpi. Fold change over uninfected samples at 1 hpi is represented. *P < 0.05; **P < 0.01; ****P < 0.0001 for two-way ANOVA and Sidak’s multiple comparisons test (*n* = 10). **E–H** Effect of glucose metabolism manipulation on WNV infection. Vero cells were infected with WNV (MOI of 1 PFU/cell) and subjected to glucose depletion (**E**), treatment with 10 µM AL-429 (**F**), 10 mM 2-DG (**G**) or 50 mM oxamate (**H**) and virus yield was determined at 24 hpi by plaque assay. Cell viability was evaluated in uninfected cells treated in parallel by quantification of cellular ATP. **P < 0.01, ***P < 0.001; ****P < 0.0001 for unpaired t-test applying Welch’s correction when differences between variances were found (*n* = 4–6)

WNV is a biosafety level 3 (BSL-3) pathogen, so biosafety concerns complicate real-time bioenergetics studies with live virus. Therefore, here, reporter virus particles (RVPs) were used as a surrogate model of infection. These RVPs constitute a single-round infectious system to study WNV infection under BSL-2 conditions [19]. RVPs were produced by *trans* complementation of a subgenomic WNV replicon with an expression vector encoding WNV structural proteins [19] (Additional file 1). Following infection with the RVPs, the expression of the reporter GFP included in the subgenomic WNV replicon was detected by fluorescence microscopy, revealing viral replication at 48 h post-infection (pi) (Additional file 1). Changes in real-time host cellular bioenergetics during infection were monitored at 1 and 48 hpi infection using a Seahorse XF analyzer. The oxygen consumption rate (OCR) was monitored to measure changes in mitochondrial respiration. No significant differences were observed between uninfected and infected cell cultures at 1 hpi (Fig. 2A). However, differences in the basal OCR were found at 48 hpi, showing a tendency to an increase in the maximal respiration and a significant increase in the spare respiratory capacity in infected cells compared to non-infected cells (Fig. 2A and B). The extracellular acidification rate (ECAR) was measured to evaluate the performance of aerobic glycolysis. No significant changes in the ECAR profiles were observed at 1 hpi, but again differences were noticed at 48 hpi (Fig. 2C) in non-glycolytic acidification and glycolysis (Fig. 2D) in infected cells. Taken together, these results support that WNV rewires host bioenergetics to augment aerobic glycolysis.

Next, the importance of glucose bioenergetics on WNV infection was explored using infectious virus. Glucose removal from the culture medium reduced virus multiplication, assessed by plaque assay of virus yield confirming the dependency on this metabolite for viral infection (Fig. 2E). AL-429, a polyphenol (Additional file 2) that inhibits glycolysis and mitochondrial respiration [18], also reduced WNV multiplication in cultured cells (Fig. 2F). To specifically address the involvement of glycolysis on WNV multiplication, the effect of reference inhibitors of the glycolytic pathway was evaluated. 2-deoxy-D-glucose (2-DG, Additional file 2), a competitive analog of glucose for hexokinase (HK), one of the rate-limiting enzymes of glycolysis, also reduced virus yield (Fig. 2G). Similarly, sodium oxamate (Additional file 2), an inhibitor of lactate dehydrogenase (LDH), a key regulator of glycolysis, lowered WNV multiplication (Fig. 2H). The impact of these treatments on cellular ATP was evaluated in uninfected cells treated in parallel. As expected for metabolic interventions targeting cellular bioenergetics, all the treatments also reduced cellular ATP content (Fig. 2E–H). These results confirmed the dependence on WNV infection on cellular bioenergetics.

### WNV infection reprograms host bioenergetics towards increased glycolysis in mouse neural tissues

**Fig. 3:**
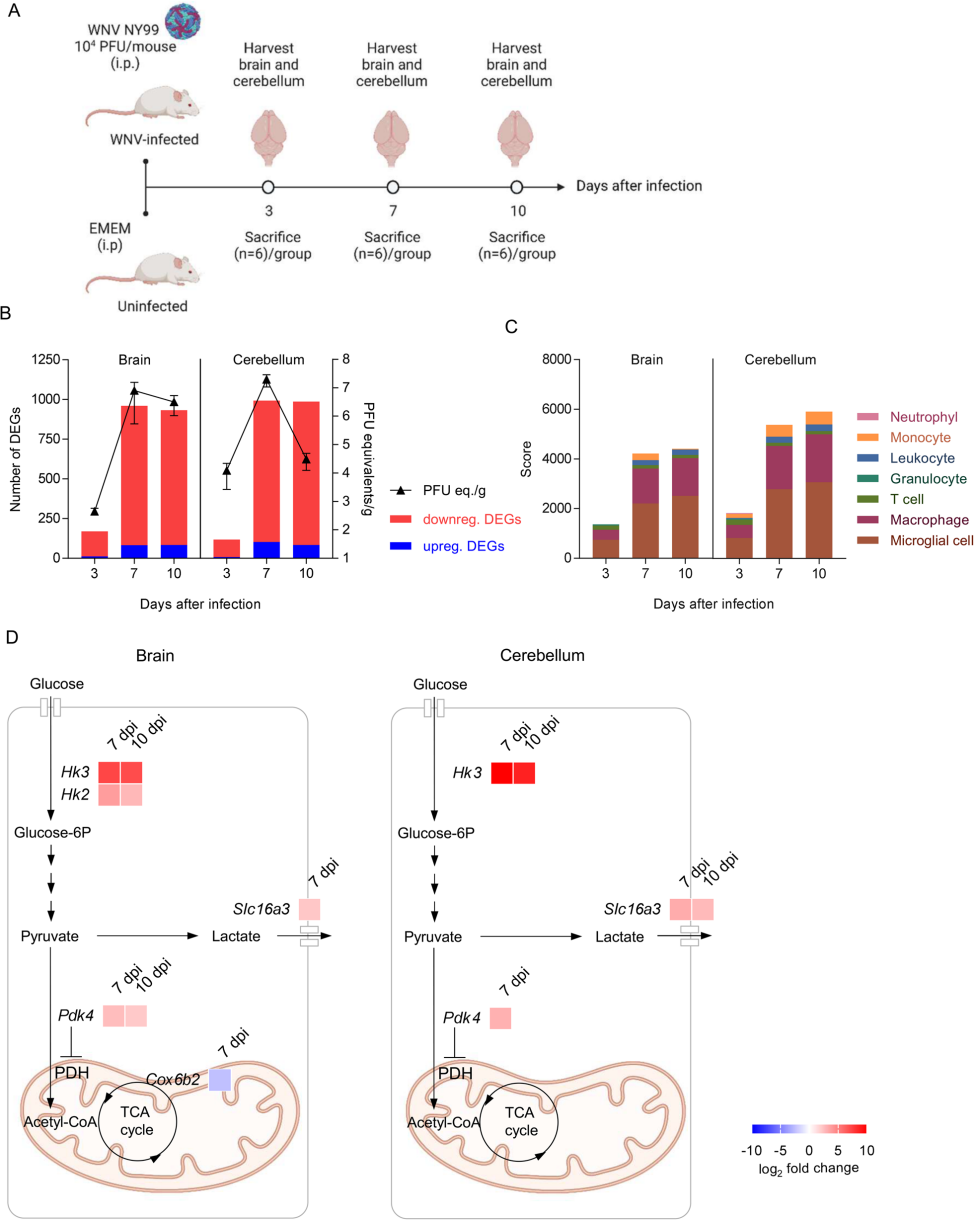
WNV infection reprograms host bioenergetics towards increased glycolysis in mouse neural tissues. **A** Experimental design and sample collection. Mice were intraperitoneally infected with 10^4^ PFU of WNV and humanly euthanized at 7 days after infection (*n* = 6). Brain and cerebellum were harvested for transcriptomic analyses. Figure was created with BioRender. **B** WNV alters the transcriptomic profile in the CNS. Virus load was quantified in the brain and cerebellum hemisphere by RT-qPCR at 3, 7 and 10 days after infection. The changes in the number of differentially expressed genes (DEGs) with |FC|> = 2 and FDR corrected *P* < 0.05 are indicated. Upregulated denotes FC > 2 and downregulated FC < 2 over uninfected animals. **C** Assessment of immune cell activation and infiltration in the brain and the cerebellum of WNV-infected mice by using bulk RNA-seq expression data to estimate the abundance of cell types during the progress of WNV neuroinvasion with CellKb. **D** DEGs related to energy metabolism in the glycolysis and oxidative phosphorylation in the brain and cerebellum of infected animals. The list of DEGs in WNV-infected tissues was filtered for genes available. Reactome pathways of glycolysis, pyruvate metabolism and TCA cycle, pentose phosphate pathway and respiratory electron transport to identify energy-related DEGs. Heat maps indicate the fold change of identified DEGs at each time post-infection

The mouse model of WNV infection recapitulates multiple aspects of natural infection, including neuroinvasion and neuroinflammation [39, 40]. Therefore, this model was selected for the study of the alterations in energy metabolism in vivo. Following experimental infection, bulk RNA-sequencing (RNA-seq) was conducted to investigate transcriptional rearrangements in the CNS of infected mice. For this purpose, the mouse brain and cerebellum were analyzed at 3, 7, and 10 dpi (Fig. 3A). The number of differentially expressed genes (DEGs) that met the criteria |log_2_ fold change|> 2 and adjusted *P*-value < 0.05 corrected by false discovery rate (FDR) increased at 7 and 10 dpi in brain and cerebellum, times pi with marked neuroinvasion as denoted by viral loads (Fig. 3B). Functional analysis of Gene Ontology (GO) terms for Biological Processes (BP) indicated that this transcriptional shift was mostly biased to immune and antiviral responses (Additional file 3). Cell marker expression analyses supported the increase in the presence/activation of immune cells in infected brains, mainly microglia, macrophages, monocytes, and leukocytes (Fig. 3C) which is consistent with previous reports describing the activation of microglia and the recruiting and infiltration of immune cells [40]. The search of a total of 163 individualized metabolic genes from the Reactome Pathway Database [31] covering glycolysis, pyruvate metabolism, TCA cycle, pentose phosphate pathway, and respiratory electron transport led to only 5 differentially expressed genes (DEGs) in infected brains (*Hk2, Hk3, Slc16a3, Pdk4,* and *Cox6b2*), being 3 of them also altered in the infected cerebellums (*Hk3, Slc16a3,* and *Pdk4*) (Fig. 3D). These energy metabolism DEGs included those related with key glycolytic enzymes *Hk2* and *Hk3* which were upregulated at 7 and 10 dpi in the brain, being *Hk3* also elevated in the cerebellum at 7 and 10 dpi. Hks control the first rate-limiting step of glucose catabolism by phosphorylating glucose to glucose-6-phosphate, fueling glycolysis. The expression of *Slc16a3* (Solute Carrier Family 16 Member 3) also known as Monocarboxylate transporter 4 (MCT4) was also upregulated at 7 dpi in the brain and a 7 and 10 dpi in the cerebellum. This membrane transporter is specialized in the export of lactate produced by glycolysis and is essential for immune metabolic reprogramming to sustain an activated inflammatory response [41]. The expression of pyruvate dehydrogenase kinase 4 (*Pdk4*), which promotes glycolysis due to inhibition of pyruvate decarboxylase complex [42], was also upregulated at 7 and 10 dpi in the brain and at 7 dpi in the cerebellum (Fig. 3D). In the case of genes related to respiratory electron transport, the Cytochrome C Oxidase Subunit 6B2 (*Cox6b2*) was repressed at 7 dpi in the brain. *Cox6b2* encodes a component of cytochrome c oxidase, the last enzyme in the mitochondrial electron transport chain which drives oxidative phosphorylation. Depletion of Cox6b2 reduces mitochondrial respiration [43], so its reduction is consistent with a redirection of energetics towards glycolysis. Gene Set Enrichment Analysis (GSEA) [28, 29] using the hallmark gene sets, which summarize and represent specific well-defined biological states or processes and display coherent expression from Mouse MSigDB Collections [30], also indicated an enrichment in the glycolytic pathway in the brain of infected animals at 7 days pi (Additional file 4). The expression of glycolytic enzymes varies across cell types in the mouse brain [44]. The data available at Brain RNA-seq resource (https://www.brainrnaseq.org/) support that *Hk3* and *Slc16a3* are more expressed by immune cells, i.e., microglia/macrophages, than by any other neural cell type in mouse brain. Therefore, the increase in its expression upon WNV infection would more probably be related to microglia activation and/or immune cell infiltration during WNV encephalitis [45–47], rather than to direct viral infection of neuronal cells. Accordingly, when the changes in the expression of these two genes were evaluated after WNV infection in Neuro-2a cells, a murine cell line from neuronal origin susceptible to WNV infection, no upregulation was observed (Additional file 5). Overall, these results support that the upregulation of glycolysis-related genes in the CNS drives energetic reprogramming during WNV infection linking glycolysis to immune response.

### 2-DG and dichloroacetate (DCA) alleviate neuroinflammation in a mouse model of WNV infection

**Fig. 4:**
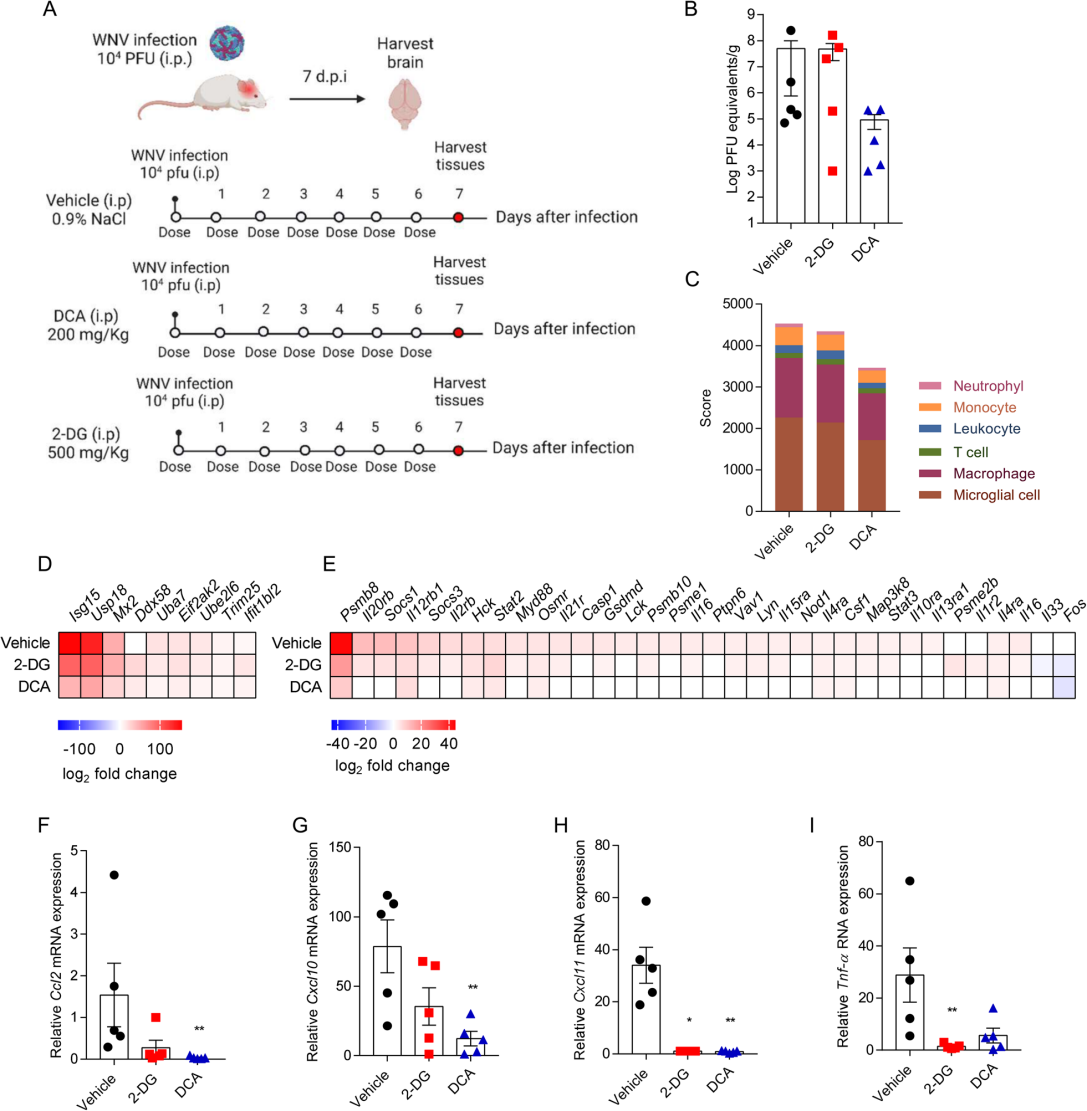
2-DG and DCA alleviate neuroinflammation in a mouse model of WNV infection. **A** Experimental design and sample collection. Mice were intraperitoneally infected with 10^4^ PFU of WNV and treated with the glycolysis inhibitors 2-DG (500 mg/kg) or DCA (200 mg/kg) diluted in saline serum by intraperitoneal injection once daily (QD) for 7 days, starting on day 0 after infection. Control mice were treated with saline serum. Animals were humanely euthanized at 7 days after infection (*n* = 5). Figure was created with BioRender. **B** WNV viral burden in the brain. Virus load was quantified in the brain by RT-qPCR. **C** Assessment of immune cell activation and infiltration in the brain of WNV-infected mice by using bulk RNA-seq expression data to estimate the abundance of cell types during the progress of WNV neuroinvasion with CellKb tool (*n* = 5 for 2-DG and DCA and *n* = 3 for vehicle). **D**, **E** Effect of the treatment with 2-DG or DCA on the DEGs related to the antiviral mechanism by IFN-stimulated genes (**D**) and signaling by interleukins (**E**) in the brain of infected animals (*n* = 5 for 2-DG and DCA and *n* = 3 for vehicle). **F–I** Cytokine induction in the brain of infected mice assessed by RT-qPCR: **E** *Ccl2*; **F** *Cxcl10*; **G** *Cxcl11*; H *Tnf-α*. *p < 0.05; **p < 0.01; for Kruskal–Wallis test and Dunn’s correction for multiple comparisons (**F**, **H**, **I**) or one-way ANOVA and Dunnett’s correction for multiple comparison (**G**) (*n* = 5)

To explore whether therapeutic intervention at the glycolytic pathway may affect WNV infection, infected mice were treated with reference inhibitors of glycolysis (Fig. 4A). The Hk inhibitor 2-DG and the Pdk4 inhibitor dichloroacetic acid (DCA) were selected based on their well-characterized targets and their reported capacity to penetrate the CNS [48, 49]. According to previous reports for the therapeutic use of these inhibitors in mice pathologies, including those of the CNS such as glioblastoma and neuroblastoma, a dose of 200 mg/kg for DCA [50, 51] and 500 mg/kg for 2-DG [48, 52] were selected. The high lethality of WNV NY99 infection in mouse model makes that, as evidenced by other authors, the effective reduction of the inflammation in the CNS of WNV-infected mice does not always correlate with a reduction in the lethality [46, 53], and in some cases it only delays the death of the animals [54]. Therefore, for this proof-of-concept study, and in order to reduce the severity of the experiments, we decided to better analyze the direct effects on the immune response of glycolysis inhibitors at 7 dpi rather than to evaluate the outcome. Brains from animals treated with DCA displayed a tendency towards lower viral loads, which was not so marked in animals treated with 2-DG (Fig. 4B). Cell marker expression analyses from data obtained by RNA-seq supported a slight reduction in the amount/activation of immune cells in animals treated with 2-DG which was more evident in mice treated with DCA (Fig. 4C). Treatment with 2-DG or DCA reduced the level of the DEGs related to antiviral mechanism by IFN-stimulated genes (mainly *Isg15*, *Usp18* and *Mx2*) in the brain of infected animals, being again more marked for DCA (Fig. 4D). Treatment with either 2-DG or DCA also reduced the expression of DEGs related to signaling by interleukins (Fig. 4E). The immunomodulatory effect of 2-DG and DCA was further confirmed by the reduction in the expression levels, assessed by quantitative RT-PCR, of genes encoding proinflammatory cytokines and chemokines that had been previously related to CNS inflammatory responses during WNV infection [54–57] such as *Ccl2* (Fig. 4F), *Cxcl10* (Fig. 4G), *Cxcl11* (Fig.H) and *Tnf*-α (Fig. 4I). Overall, these data suggest that the treatment with glycolysis inhibitors can alleviate WNV-induced neuroinflammation. These results are in agreement with the recently proposed approach of targeting immunometabolism as an antiviral and anti-inflammatory strategy [6] and support the potential of compounds affecting the glycolytic pathway such as 2-DG to combat viral diseases [58]. However, the poor pharmacokinetic properties of 2-DG will probably limit its wide clinical application and the development of improved analogues is required [58]. On the other hand, DCA has favorable pharmacokinetics and has previously shown therapeutic efficacy for the treatment of lactic acidosis in children [59] and at preclinical stages in several cancer models [60] making it an interesting candidate for drug repositioning against viral diseases. In summary, our results support the induction of a metabolic shift in target tissues towards glycolysis during WNV infection. Moreover, pharmacological intervention at the glycolysis pathway reduced neuroinflammation in mice models of WNV infection. These results open new perspectives for the future development of effective therapies to combat WNV disease relying on immunometabolism modulators.

### Supplementary Information

**Additional file 1. ** WNV RVPs production and infection. (A) WNV single-round reporter virus particles (RVPs) were produced by co-transfection of HEK 293 T cells with a subgenomic reporter replicon expressing GFP and a plasmid expressing WNV structural proteins C, prM and E. The RVPs were collected from the supernatants at 48 h post-transfection to infect Vero cells monolayers for real-time bioenergetic analyses. Infection was confirmed by using a fluorescence microscope to detect infected green fluorescent cells. Figure was created with BioRender. (B) Fluorescence micrographs of Vero cells infected with RVPs or not (negative control) at 48 h after infection.

**Additional file 2.** Chemical structures of the metabolic inhibitors used in the study. Structures of Compound AL-429, 2-DG, sodium oxamate and DCA.

**Additional file 3. **Functional annotation of top 8 Gene Ontology Biological Process (GO BP) DEGs in brain and cerebellum. Bubble plots showing enrichment value (-log10 *p*-value) and gene ratio for each GO BP term were created by using SRplot (https://www.bioinformatics.com.cn/srplot). (A) Top 8 GO BP for WNV-infected brain DEGs at 3, 7 and 10 dpi. (B) Top 8 GO BP for WNV-infected cerebellum DEGs at 3, 7 and 10 dpi.

**Additional file 4. ** GSEA enrichment plot of glycolysis in brains derived from WNV-infected mice at 7 dpi. GSEA was performed against the hallmark gene set database from Mouse MSigDB Collections. Glycolysis gene set was significantly and positively enriched in the brains of WNV-infected mice. Enrichment score (ES) is represented in the y-axis and as a green curve and it represents the degree of over-representation of a gene set of the ranked gene list. Positive or negative correlation of genes with WNV infection phenotype is shown at the colored band at the bottom (red for positive and blue for negative correlation). Significance FDR threshold was at < 0.05. Normalized enrichment score (NES) and FDR corrected q-value are indicated.

**Additional file 5. **Infection with WNV does not upregulate Hk3 and Slc16a3 expression in neuronal cells. Neuro-2a cells were infected with WNV (MOI of 1 PFU/cell) and the expression of *Hk3* (A) and *Slc16a3* (B) was determined by quantitative RT-PCR relative to that of *GAPDH* at 24 hpi. Samples from infected and uninfected mouse brains at 10 dpi were also included to allow direct comparison. Two-way ANOVA and Sidak’s multiple comparison tests were performed (*n* = 3). ****P < 0.0001.

## Data Availability

RNA-seq datasets generated in this work were uploaded to GEO (https://www.ncbi.nlm.nih.gov/geo/) under Accession GSE233218.

## Abbreviations

BSL: Biosafety level
CNS: Central nervous system
Cox6b2: Cytochrome C Oxidase Subunit 6B2
CSF: Cerebrospinal fluid
DCA: Sodium dichloroacetate
DEG: Differentially expressed gene
2-DG: 2-Deoxy-D-glucose
DMSO: Dimethyl sulfoxide
dpi: Days post-infection
ECAR: Extracellular acidification rate
FCCP: Trifluoromethoxy carbonylcyanide phenylhydrazone
FC: Fold change
FDR: False discovery rate
GSEA: Gene Set Enrichment Analysis
Hk: Hexokinase
LDH: Lactate dehydrogenase
OCR: Oxygen consumption rate
Pdk4: Pyruvate dehydrogenase kinase 4
RVP: Reporter virus particle
TCA: Tricarboxylic acid
Slc16a3: Solute Carrier Family 16 Member 3
WNV: West Nile virus
