# Supplementary material for: Glycolytic shift during West Nile virus infection provides new therapeutic opportunities
Source: J Neuroinflammation. 2023 Sep 27;20:217. doi: 10.1186/s12974-023-02899-3 (PMC10537838; doi:10.1186/s12974-023-02899-3)
Supplement: Supplementary file 1 — Additional file 1. WNV RVPs production and infection. (A) WNV single-round reporter virus particles (RVPs) were produced by co-transfection of HEK 293 T cells with a subgenomic reporter replicon expressing GFP and a plasmid expressing WNV structural proteins C, prM and E. The RVPs were collected from the supernatants at 48 h post-transfection to infect Vero cells monolayers for real-time bioenergetic analyses. Infection was confirmed by using a fluorescence microscope to detect infected green fluorescent cells. Figure was created with BioRender. (B) Fluorescence micrographs of Vero cells infected with RVPs or not (negative control) at 48 h after infection. [file 12974_2023_2899_MOESM1_ESM.pdf]

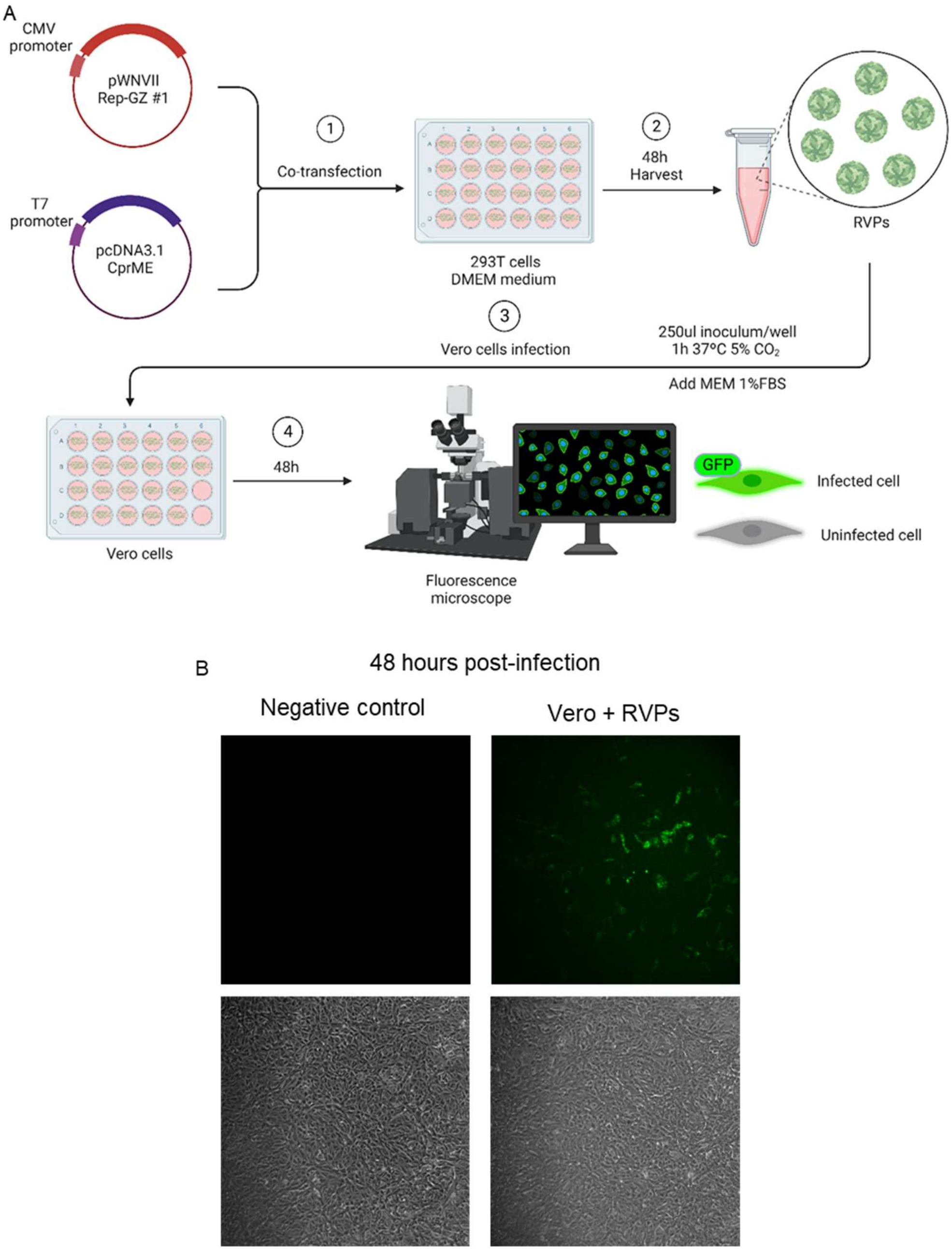

Additional file 1. WNV RVPs production and infection. (A) WNV single-round reporter virus particles (RVPs) were produced by co-transfection of HEK 293T cells with a subgenomic reporter replicon expressing GFP and a plasmid expressing WNV structural proteins C, prM and E. The RVPs were collected from the supernatants at 48 h post-transfection to infect Vero cells monolayers for real-time bioenergetic analyses. Infection was confirmed by using a fluorescence microscope to detect infected green fluorescent cells. Figure was created with BioRender. **(B)** Fluorescence micrographs of Vero cells infected with RVPs or not (negative control) at 48 h after infection.
