# Supplementary material for: Glycolytic shift during West Nile virus infection provides new therapeutic opportunities
Source: J Neuroinflammation. 2023 Sep 27;20:217. doi: 10.1186/s12974-023-02899-3 (PMC10537838; doi:10.1186/s12974-023-02899-3)
Supplement: Supplementary file 2 — Additional file 2. Chemical structures of the metabolic inhibitors used in the study. Structures of Compound AL-429, 2-DG, sodium oxamate and DCA. [file 12974_2023_2899_MOESM2_ESM.pdf]

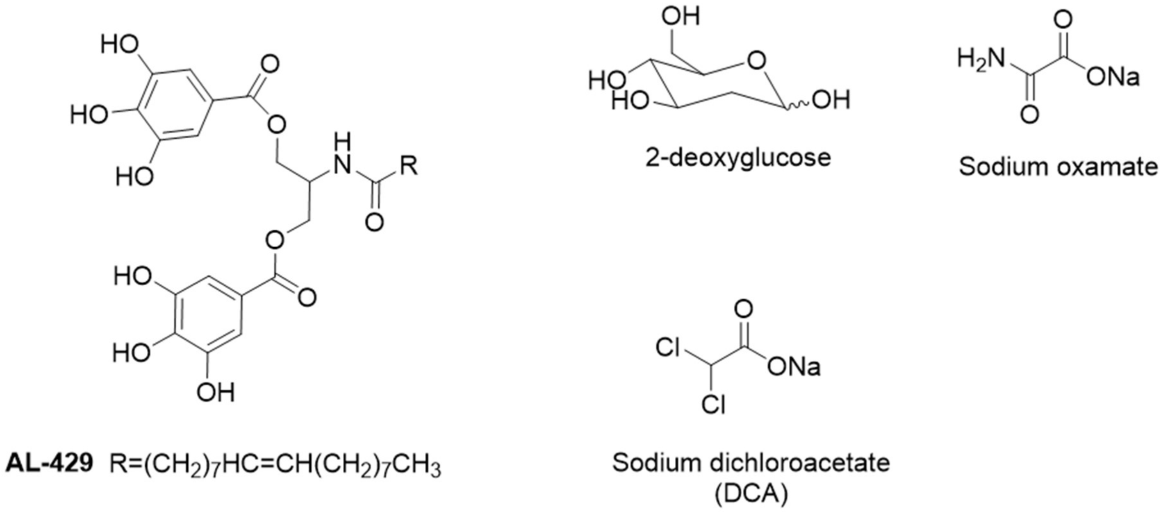

Additional file 2. Chemical structures of the metabolic inhibitors used in the study. Structures of Compound AL-429, 2-DG, sodium oxamate and DCA.
