# Supplementary material for: Glycolytic shift during West Nile virus infection provides new therapeutic opportunities
Source: J Neuroinflammation. 2023 Sep 27;20:217. doi: 10.1186/s12974-023-02899-3 (PMC10537838; doi:10.1186/s12974-023-02899-3)
Supplement: Supplementary file 3 — Additional file 3. Functional annotation of top 8 Gene Ontology Biological Process (GO BP) DEGs in brain and cerebellum. Bubble plots showing enrichment value (-log10 p-value) and gene ratio for each GO BP term were created by using SRplot (https://www.bioinformatics.com.cn/srplot). (A) Top 8 GO BP for WNV-infected brain DEGs at 3, 7 and 10 dpi. (B) Top 8 GO BP for WNV-infected cerebellum DEGs at 3, 7 and 10 dpi. [file 12974_2023_2899_MOESM3_ESM.pdf]

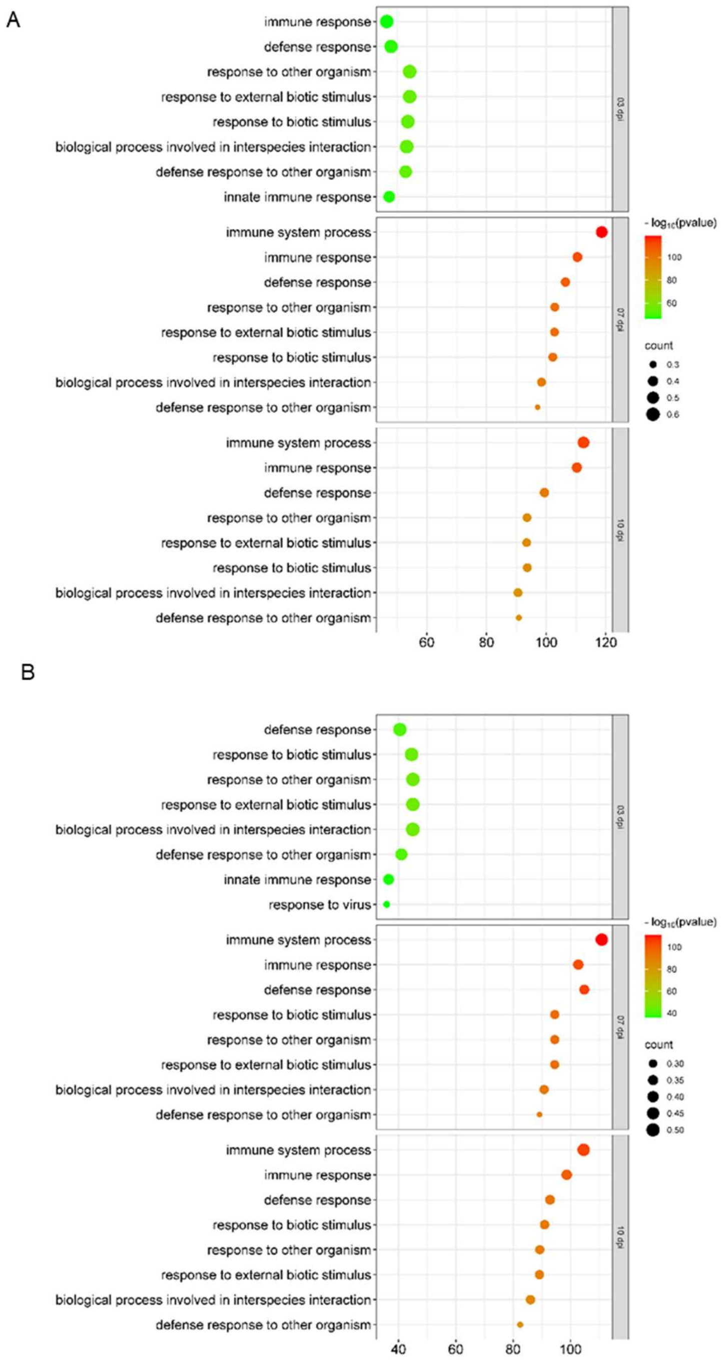

Additional file 3. Functional annotation of top 8 Gene Ontology Biological Process (GO BP) DEGs in brain and cerebellum. Bubble plots showing enrichment value (-log10 p-value) and gene ratio for each GO BP term were created by using SRplot (https://www.bioinformatics.com.cn/srplot). **(A)** Top 8 GO BP for WNV-infected brain DEGs at 3, 7 and 10 dpi. **(B)** Top 8 GO BP for WNV-infected cerebellum DEGs at 3, 7 and 10 dpi.
