# Supplementary material for: Glycolytic shift during West Nile virus infection provides new therapeutic opportunities
Source: J Neuroinflammation. 2023 Sep 27;20:217. doi: 10.1186/s12974-023-02899-3 (PMC10537838; doi:10.1186/s12974-023-02899-3)
Supplement: Supplementary file 4 — Additional file 4. GSEA enrichment plot of glycolysis in brains derived from WNV-infected mice at 7 dpi. GSEA was performed against the hallmark gene set database from Mouse MSigDB Collections. Glycolysis gene set was significantly and positively enriched in the brains of WNV-infected mice. Enrichment score (ES) is represented in the y-axis and as a green curve and it represents the degree of over-representation of a gene set of the ranked gene list. Positive or negative correlation of genes with WNV infection phenotype is shown at the colored band at the bottom (red for positive and blue for negative correlation). Significance FDR threshold was at < 0.05. Normalized enrichment score (NES) and FDR corrected q-value are indicated. [file 12974_2023_2899_MOESM4_ESM.pdf]

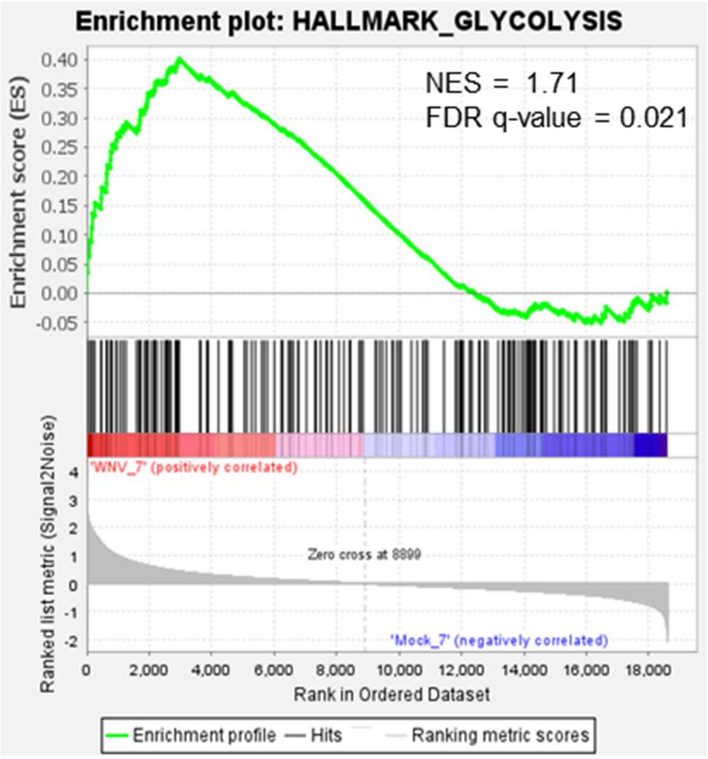

Additional file 4. GSEA enrichment plot of glycolysis in brains derived from WNV-infected mice at 7 dpi. GSEA was performed against the hallmark gene set database from Mouse MSigDB Collections. Glycolysis gene set was significantly and positively enriched in the brains of WNV-infected mice. Enrichment score (ES) is represented in the y-axis and as a green curve and it represents the degree of over-representation of a gene set of the ranked gene list. Positive or negative correlation of genes with WNV infection phenotype is shown at the colored band at the bottom (red for positive and blue for negative correlation). Significance FDR threshold was at < 0.05. Normalized enrichment score (NES) and FDR corrected q-value are indicated.
