# Supplementary material for: Glycolytic shift during West Nile virus infection provides new therapeutic opportunities
Source: J Neuroinflammation. 2023 Sep 27;20:217. doi: 10.1186/s12974-023-02899-3 (PMC10537838; doi:10.1186/s12974-023-02899-3)
Supplement: Supplementary file 5 — Additional file 5. Infection with WNV does not upregulate Hk3 and Slc16a3 expression in neuronal cells. Neuro-2a cells were infected with WNV (MOI of 1 PFU/cell) and the expression of Hk3 (A) and Slc16a3 (B) was determined by quantitative RT-PCR relative to that of GAPDH at 24 hpi. Samples from infected and uninfected mouse brains at 10 dpi were also included to allow direct comparison. Two-way ANOVA and Sidak’s multiple comparison tests were performed (n = 3). ****P < 0.0001. [file 12974_2023_2899_MOESM5_ESM.pdf]

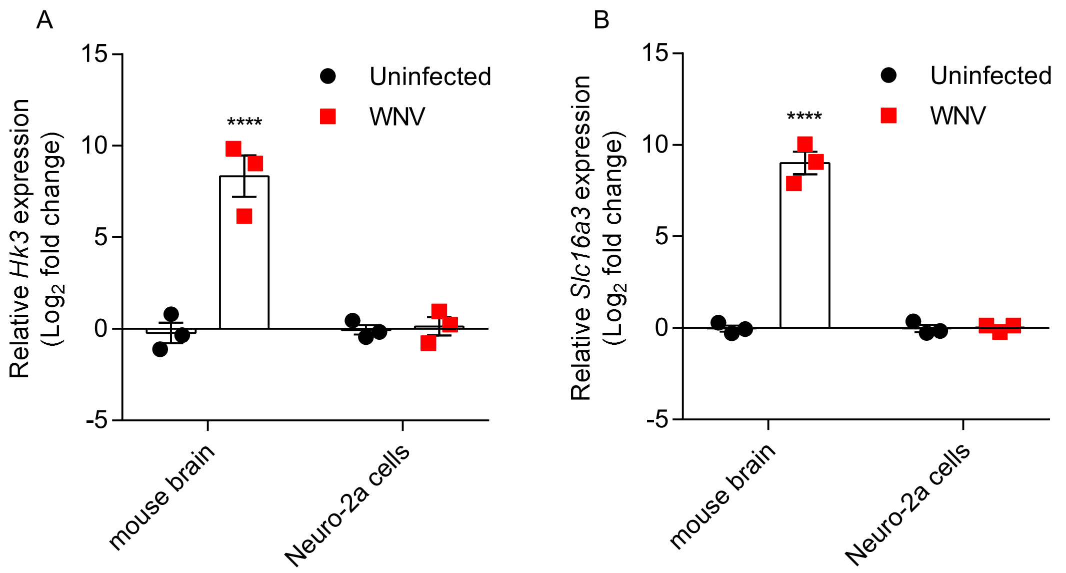

Additional file 5. Infection with WNV does not upregulate *Hk3* and *Slc16a3* expression in neuronal cells. Neuro-2a cells were infected with WNV (MOI of 1 PFU/cell) and the expression of *Hk3* **(A)** and *Slc16a3* **(B)** was determined by quantitative RT-PCR relative to that of *GAPDH* at 24 hpi. Samples from infected and uninfected mouse brains at 10 dpi were also included to allow direct comparison. Two-way ANOVA and Sidak's multiple comparison tests were performed (n=3). ****, P<0.0001.
